# Touch Avoidance with Close People and Strangers: Effects of Gender, Sexual Orientation, and Relationship Status

**DOI:** 10.3390/ejihpe13090134

**Published:** 2023-09-14

**Authors:** Francesco Bruno, Chloe Lau, Carlotta Tagliaferro, Lena C. Quilty, Francesca Chiesi

**Affiliations:** 1 Regional Neurogenetic Centre (CRN), Department of Primary Care, ASP Catanzaro, Viale A. Perugini, 88046 Lamezia Terme, Italy; 2 Association for Neurogenetic Research (ARN), 88046 Lamezia Terme, Italy; 3 Academy of Cognitive Behavioral Sciences of Calabria (ASCoC), 88046 Lamezia Terme, Italy; 4 Centre for Addiction and Mental Health, Toronto, ON N6B 1Y6, Canada; lena.quilty@camh.ca; 5 School of Psychology, University of Florence, 50135 Florence, Italy; carlotta.tagliaferro@stud.unifi.it; 6 Department of Neuroscience, Psychology, Drug, and Child’s Health (NEUROFARBA), Section of Psychology, University of Florence, 50135 Florence, Italy; francesca.chiesi@unifi.it

**Keywords:** touch avoidance, Touch Avoidance Questionnaire (TAQ), gender, sexual orientation, relationship status, family, partner, same-sex friends, opposite-sex friends, strangers

## Abstract

Human contact through physical touch is a core element in social bonding, which facilitates psychosocial well-being. Touch avoidance is an individual disposition that may prevent individuals from engaging in or benefiting from physical touch. The present study recruited 450 Italian participants (51.1% female) with a mean age of 32.2 ± 13.5 to complete a battery of demographic questionnaires and the Touch Avoidance Questionnaire (TAQ). Individuals who were single and reporting same-sex attraction avoided touch with family more often than their coupled counterparts or those reporting opposite-sex attraction. Moreover, males reporting same-sex attraction avoided touch with a potential partner more frequently. When comparing sex differences, women reported greater touch avoidance with opposite-sex friends more frequently, while males avoided touch with same-sex friends more frequently. Individuals reporting opposite-sex attraction reported greater touch amongst same-sex friends. Single males avoided touch with same-sex friends more frequently than those in a relationship. Overall, this contribution reflects the individual differences related to social touch avoidance with respect to sex, relationship status, and sexual orientation in an Italian sample.

## 1. Introduction

Human contact through physical touch is a core element in social bonding, which facilitates psychosocial well-being [1,2,3]. Indeed, researchers have found that positive touch, which has decreased in the context of a global pandemic, may affect psychological well-being [3]. Physical touch enables the communication of motivations and emotions, and early maternal touch is associated with the age-appropriate development of infant social brain areas and secure attachment [4,5,6,7,8]. Interestingly, the frequency of touch from one’s mother predicts resting activity and connectivity within the developing child’s social brain [4]. The most well-known hypothesis regarding the biological mechanisms of social touch is the Affective Touch Hypothesis, which postulates that the C-tactile afferents system enhances feelings of pleasure, closeness, and security [9]. Affective touch facilitates better relationships with people close to an individual, which allows for social bonding with other individuals from specific communities [9]. Individuals who received physical touch on specific days with their partners reported enhanced closeness, relationship quality, perceived partner responsiveness, and accommodative behaviors compared to days without affectionate touch [10]. Indeed, touch represents an important component of the human experience that remains necessary for greater levels of relationship satisfaction, commitment, and conflict management [11,12,13,14]. Furthermore, touch may enhance social experiences for individuals, as gestures, such as handshakes, may indicate feelings of trust and gratitude towards the individual [7,15].

Given the importance of affective physical contact with other people, measuring attitudes towards touch is essential in helping individuals build relationships with family, friends, and acquaintances, while also enabling patients and their healthcare practitioners develop better working relationships [16]. In particular, touch avoidance, which is defined as the active or passive avoidance of touch from other individuals, including one’s family members, friends, and acquaintances, has been thoroughly investigated in the past decade in determining the impact of non-verbal communication evasion within intimate relationships [16,17,18]. Thus, touch avoidance is associated with the individual’s willingness to touch others, which may impact how an individual perceives touch and how much touch the individual gets in return [18].

Data suggest that touch avoidance is linked with homophily and interpersonal attraction, as touch avoidance is associated with feeling less attraction and homophily towards individuals who touch them [19]. Sex roles also play an important part in touch avoidance, as males may be more likely to have same-sex touch avoidance compared to females [20,21,22,23]. These findings may be attributed to social norms, given that males initiating and facilitating touch may be less common and perceived negatively compared to females initiating touch [20,21,22,23]. Individuals scoring higher in androgyny indicated less same-sex and opposite-sex touch avoidance compared to those low in androgyny [20]. Across over 3000 participants in 40 universities, Andersen and Leibowitz found that opposite-sex touch avoidance was higher for females than males and that this disposition was negatively associated with an open communication style and self-esteem [17]. Cultural differences may also be implicated within same-sex and opposite-sex touch avoidance patterns [24]. Additionally, individual differences that affect an individual’s threshold for sensory inputs (e.g., autistic traits) may be at play [25]. Individual differences and characteristics should be considered when investigating the roles of touch avoidance.

Moreover, Russo and colleagues [26] noted that sex differences in the discernment of positive touch have not been thoroughly researched. These researchers also reported that women welcomed physical touch and have a more pleasurable experience with touch compared to men [26]. Consistent with these findings, women reported more experiences of physical touch in their day-to-day interactions compared to men [27,28]. While women are often the recipients of touch, men tend to initiate touch especially if the recipient is a woman [29,30]. Moreover, men reported preference with being touched by women than by men [31,32]. Men also indicated greater touch avoidance with their partner, family, and same-sex individuals compared to women [33]. These sex differences may account for individual differences in preference and frequency in physical touch [26]. The recipient and provider of touch should be considered to thoroughly understand the responses associated with touch avoidance.

Although studies have investigated the roles of sex differences, to our knowledge, no studies have investigated the effects of relationship status and sexual orientation in touch avoidance with family members, partners, same- and opposite-sex friends, and strangers.

The present study seeks to investigate specific research questions on individual differences in experiences of positive touch. Firstly, it is unclear whether preference or avoidance for touch is impacted by cultural differences in the perception of the appropriateness of touch across different situations. Specifically, the present study aims to establish whether previous findings are replicable in an Italian sample. Secondly, men and women reporting same-sex attraction may also experience differential effects in physical touch avoidance than men and women reporting opposite-sex attraction. Finally, physical touch was most common amongst romantic partners and children across cultures. Not surprisingly, individuals enable more physical touch in their close relationships [8,34]. The aim of this study was to analyze the effects of self-identified gender, sexual orientation, and relationship status in social touch avoidance with close people (i.e., a potential partner, family members, same-sex friends, opposite-sex friends) and strangers among Italian people.

## 2. Materials and Methods

### 2.1. Participants and Procedure

As part of a large study on the Italian general population, all participants meeting the following criteria, based on self-report, were included in this sample: 1. Italian citizens; 2. adults (i.e., age 18 and older); 3. have stated their gender as male or female, their sexual orientation as same-sex or opposite-sex attraction, their relationship status as single or in a relationship; 4. no mental health diagnoses; 5. not currently taking psychiatric medication; 6. all questions administered in the questionnaires were completed. The final sample was composed of 450 participants (51.1% female) with a mean age of 32.2 ± 13.5, ranging from 18 to 71 years. Most participants reported being attracted to the opposite sex (77.2% male and 77% female, respectively), in a relationship (59.3%), had a high school education ≤ 13 years (64%), and were unemployed (55.1%) (Table 1). The study was a web-based survey designed to involve participants in all Italian regions. The survey was developed using Google Forms^®^ and was distributed through social networking sites such as Facebook, WhatsApp, and Instagram using sponsored social network advertisements together with a snowball recruiting technique between May and June 2021. Participants were informed that their participation was voluntary, their responses would remain anonymous, and they could withdraw from the survey at any time without penalty. All participants signed an online informed consent and did not receive an incentive. The research protocol was approved by the Ethical Committee of Regione Calabria—Area Centrale (Catanzaro, Italy).

### 2.2. Measures

***Socio-demographics characteristics.*** Participants reported their age, gender, sexual orientation, educational level, relationship status, and employment status within the survey.

**Touch Avoidance Questionnaire (TAQ).** The Italian version of the TAQ instrument [16] was used to assess attitudes toward touch in different types of relations. It is composed of 31 items rated on a 5-point Likert scale ranging from “*fully disagree*” to “*fully agree*”. The scores of all items are added together for each of the 5 subscales (i.e., partner, family, same-sex friends, opposite-sex friends, and strangers); higher sub-scores indicated higher avoidance for that particular type of social touch. In our sample, the Cronbach’s α was 0.87 for partner, 0.78 for family, 0.84 for same-sex friends, 0.85 for opposite-sex friends, and 0.56 for strangers’ subscales.

### 2.3. Statistical Analysis

Data were analyzed in Jamovi software (version 2.3.18). Descriptive statistics were conducted on demographic characteristics. Means and standard deviations for continuous variables and frequencies and percentages of categorical variables were generated. Analysis of covariance (ANCOVA), controlled for age, was conducted to test the effects and the interactions of gender (male/female), sexual orientation (same-sex or opposite-sex attraction), and relationship status (single/in relationship) on social touch avoidance with family members, partner, same- and opposite-sex friends, and strangers.

## 3. Results

The results of the ANCOVA analysis controlled for age are shown in Table 2. Regarding touch avoidance with family members (i.e., parents, siblings), the analysis showed a significant main effect of sexual orientation (*F* = 11.807, df = 1, *p* = <0.001, *η^2^p* = 0.026), indicating that individuals reporting same-sex attraction avoided touch with family members (estimated marginal mean (EMM) opposite-sex attraction = 15.7 ± 0.32; EMM same-sex attraction = 18 ± 0.59). Of the sexual orientation/relationship status interaction (*F* = 4.816, df = 1, *p* = 0.029, *η*^2^*p* = 0.011), individuals with same-sex attraction who were single avoided touch with family more often (EMM opposite-sex attraction single = 15.2 ± 0.52; EMM opposite-sex attraction in a relationship = 16.1 ± 0.41, EMM same-sex attraction single = 19.0 ± 0.82, EMM same-sex attraction in a relationship = 16.9 ± 0.83).

Regarding touch avoidance with a potential partner, the analysis showed a significant main effect of relationship status (*F* = 13.430, df = 1, *p* < 0.001, *η*^2^*p* = 0.025), indicating that single individuals avoided touch more with a potential partner (EMM single = 20.4 ± 0.65; EEM in a relationship = 17.1 ± 0.60). Of the gender/sexual orientation interaction (*F* = 9.773, df = 1, *p* = 0.002, *η*^2^*p* = 0.022), males who identified as experiencing same-sex attraction avoided touch with a potential partner more frequently (EMM opposite-sex attraction male = 17.9 ± 0.59; EMM opposite-sex attraction female = 19.3 ± 0.61, EMM same-sex attraction male = 20.9 ± 1.10, EMM same-sex attraction female = 16.8 ± 1.07).

Concerning the touch avoidance with same-sex friends, the analysis showed a significant main effect of gender (*F* = 30.420, df = 1, *p* < 0.001, *η*^2^*p* = 0.065). Results indicate that males avoided touch with same-sex friends (EMM male = 13.04 ± 0.41; EMM female = 9.80 ± 0.41). The main effect of sexual orientation (*F* = 15.907, df = 1, *p* < 0.001, *η*^2^*p* = 0.035) suggests that opposite-sex attraction was linked with more touch amongst same-sex friends (EMM opposite sex attraction = 12.6 ± 0.28; EMM same sex attraction = 10.2 ± 0.52). The gender/sexual orientation interaction (*F* = 3.888, df = 1, *p* = 0.049, *η*^2^*p* = 0.009) suggests that males reporting opposite-sex attraction avoided touch of same-sex friends more frequently (EMM opposite-sex attraction male = 14.81 ± 0.39; EMM opposite-sex attraction female = 10.40 ± 0.41, EMM same-sex attraction male = 11.28 ± 0.74, EMM same-sex attraction female = 9.19 ± 0.72). The gender/relationship status interaction (*F* = 8.241, df = 1, *p* = 0.004, *η*^2^*p* = 0.018) reflects that single males avoided touch with same-sex friends more frequently than those in a relationship (EMM male single = 14.10 ± 0.56; EMM male in a relationship = 11.98 ± 0.63, EMM female single = 9.16 ± 0.64, EMM female in a relationship = 10.43 ± 0.51). The gender/sexual orientation/ relationship status interaction was also significant (*F* = 6.952, df = 1, *p* = 0.009, *η*^2^*p* = 0.016). Specifically, males who reported opposite-sex attraction and being single avoided touch more with same-sex friends most often. The EMMs of the different subgroups are reported in Table 3.

With regard to touch avoidance with opposite-sex friends, the analysis showed a significant main effect of gender (*F* = 7.660, *p* = 0.006, *η*^2^*p* = 0.017), indicating that females avoided touch with opposite-sex friends more frequently (EMM male = 11.3 ± 0.46; EMM female = 13.1 ± 0.46).

Finally, touch avoidance with strangers yielded no significant results from the analysis carried out.

## 4. Discussion

While previous research has emphasized the importance of gender differences, this study has further extended previous findings by investigating how touch avoidance may differ with opposite-sex friends, family members, and partners based on the individual’s gender and self-identified sexual orientation. Notably, Passarelli et al. [22] noted same-sex friends touch avoidance, which was replicated in this study. Several interesting findings emerged in the present study. Individuals reporting same-sex attraction who were single avoided touch with family more often than their coupled counterparts or those reporting opposite-sex attraction. Moreover, males reporting same-sex attraction avoided touch with a potential partner more frequently. Floyd found that perceived homophobia was negatively associated to evaluations of same-sex affectionate touch [35]. Researchers and clinicians may benefit from understanding this research in order to explore touch initiation and avoidance for individuals with same-sex attraction.

Single individuals reporting same-sex attraction also avoided touch with family members more frequently compared to their opposite-sex attracted counterparts. Moreover, individuals reporting opposite-sex attraction indicated greater touch amongst same-sex friends. Single males avoided the touch of same-sex friends more frequently than those in a relationship. These results highlight the importance of noting the recipient and the actor of a physical touch interaction. Minority stress theory may provide a plausible account for this finding. Minority stress theory postulates that stigma-related stress that is initiated by chronic stressors may negatively impact sexual minority individuals, whether it be distal or proximal in nature [36,37,38]. Brandt and colleagues [39] have reported internalized heterosexism as a barrier to intimacy, predicting that lower engagement in intimate behaviors and internalized stigma may also impact an individual’s comfort with intimacy [40]. While individuals may desire touch, internalized heterosexism may decrease the probability that an individual engages in positive touch as it may enhance perceived stigma and discrimination [40]. Indeed, physical touch avoidance may reflect difficulties sexual minority individuals face that may not be experienced by their heterosexual counterparts. These results also stress the need to explore intersectional identity when investigating experiences of social touch amongst people [41].

When comparing sex differences, women reported greater touch avoidance with opposite-sex friends more frequently, while males avoided touch with same-sex friends more frequently. These results are in line with previous findings, demonstrating that women are often the recipients of touch, whereas men tend to initiate touch, especially if the recipient is a woman [29,30]. Moreover, men reported preference for being touched by women rather than other men [31,32]. Men also noted greater touch avoidance with their partner, family, and same-sex individuals compared to women [33]. The results are consistent with other findings in previous studies [26].

There are significant strengths to the present study. Specifically, a large number of Italians were recruited with different sexual orientation, relationship status, age, and sex. While many previous findings recruited from university samples, the present study has a larger representation with individuals from different age groups. Moreover, the present research questions had not been investigated in an Italian sample, although there is one other study that investigated some different research questions [22]. It is important to replicate findings regarding positive touch across different ages, cultures, and demographics. There are specific cultural differences with respect to the perception of touch between different settings that may have diverse rules governing appropriate affect, behavior, and cognition. Research regarding touch initiation and avoidance would benefit from replication across various cultures to study these differences. Despite the large representative sample size, there are several limitations in this study. First, the measures were completed based on self-reporting and may be limited based on insight and desirability biases. Future studies may corroborate current findings with peer-reporting or observational studies. Second, Casetta et al. [16] reported that the stranger subscale has lower internal consistency and the present study replicated these findings. It may be beneficial for future scales to specify the meaning of stranger (e.g., an individual may react differently if a friend of a friend-initiated touch as opposed to a stranger in a novel setting). Moreover, there may be specific cultural contexts where beliefs, attitudes, approaches, behaviors, and social output towards strangers may differ. Some cultures may welcome the initiation of physical and emotional closeness with strangers, whereas other cultures may frown upon it. A more refined definition of the stranger term may yield greater reliability.

## 5. Conclusions

Overall, this paper uniquely contributes to the literature in understanding social touch avoidance with respect to sex, relationship status, and sexual orientation in an Italian sample. Future contributions may continue to extend this work through investigating the experiences of social touch in individuals with intersectional identities and may explore whether perceptions of same-sex relationships affect the associations found in the present study. In addition, subsequent research may analyze methods to enhance the internal consistency of this subscale and decrease any potential measurement error. It is important to recognize that the present findings may not be generalizable to individuals residing in other countries as they experience different cultural norms and values. Given that touch is essential for generating and solidifying social bonds, other studies should investigate whether the lower frequency of physical touch may affect the physical and psychological well-being of individuals reporting same-sex attraction. Future studies should also consider the effects of other diverse sexual orientations (e.g., bisexuality, pansexuality, asexuality, and demisexuality) and gender expressions (e.g., gender queer) on touch avoidance. Moreover, given that touch avoidance is typically assessed with self-reporting, observational studies or laboratory studies that monitor an individual’s level of touch providing quantity and quality of assessment would yield greater validity.

## Figures and Tables

**Table 1:** Sample characteristics.

**Age (Years, Mean ± SD)**	32.2 ± 13.5 Range 18–71
**Gender**	
Male	220 (48.9)
Female	230 (51.1)
**Sexual orientation**	
*Among males:*	
Opposite-Sex Attraction	170 (77.2)
Same-Sex Attraction	50 (22.8)
*Among females:*	
Opposite-Sex Attraction	177 (77)
Same-Sex Attraction	53 (23)
**Relationship Status**	
Single	183 (40.7)
In a relationship	267 (59.3)
**School Education**	
≤13 years	288 (64)
>13 years	162 (36)
**Occupation**	
Employed	202 (44.9)
Unemployed	248 (55.1)

*Note*. Data are presented as N (%) or mean ± SD.

**Table 2:** Analysis of covariance (ANCOVA) controlled for age.

	Sum of Squares	df	Mean Square	*F*	*p*-Value	*η* ^2^	*η* ^2^ *p*	ω²
**Touch Avoidance—Family**								
Gender	29.2313	1	29.2313	0.871	0.351	0.002	0.002	−0.000
Sexual orientation	396.4035	1	396.4035	11.807	<0.001 ***	0.026	0.026	0.023
Relationship status	25.1487	1	25.1487	0.749	0.387	0.002	0.002	−0.001
Gender × Sexual orientation	61.0446	1	61.0446	1.818	0.178	0.004	0.004	0.002
Gender × Relationship status	18.4491	1	18.4491	0.550	0.459	0.001	0.001	−0.001
Sexual orientation × Relatioship status	161.6972	1	161.6972	4.816	0.029 *	0.010	0.011	0.008
Gender × Sexual orientation × Relationship status	24.1312	1	24.1312	0.719	0.397	0.002	0.002	−0.001
**Touch Avoidance—** **Partner**								
Gender	138.631	1	138.631	2.37780	0.124	0.005	0.005	0.003
Sexual orientation	3.585	1	3.585	0.06149	0.804	0.000	0.000	−0.002
Relationship status	783.035	1	783.035	13.43064	<0.001 ***	0.027	0.030	0.025
Gender × Sexual orientation	569.838	1	569.838	9.77387	0.002 **	0.020	0.022	0.018
Gender × Relationship status	0.557	1	0.557	0.00956	0.922	0.000	0.000	−0.002
Sexual orientation × Relationship status	0.275	1	0.275	0.00472	0.945	0.000	0.000	−0.002
Gender × Sexual orientation × Relationship status	146.460	1	146.460	2.51209	0.114	0.005	0.006	0.003
**Touch Avoidance—** **Same-sex friends**								
Gender	795.9	1	795.9	30.420	<0.001 ***	0.059	0.065	0.057
Sexual orientation	416.2	1	416.2	15.907	<0.001 ***	0.031	0.035	0.029
Relationship status	13.2	1	13.2	0.506	0.477	0.001	0.001	−0.001
Gender × Sexual orientation	101.7	1	101.7	3.888	0.049 *	0.008	0.009	0.006
Gender × Relationship status	215.6	1	215.6	8.241	0.004 **	0.016	0.018	0.014
Sexual orientation × Relationship status	25.7	1	25.7	0.983	0.322	0.002	0.002	0.000
Gender × Sexual orientation × Relationship status	181.9	1	181.9	6.952	0.009 **	0.014	0.016	0.012
**Touch Avoidance—** **Opposite-sex friends**								
Gender	245.77	1	245.77	7.660	0.006 **	0.017	0.017	0.014
Sexual orientation	33.72	1	33.72	1.051	0.306	0.002	0.002	−0.000
Relationship status	13.60	1	13.60	0.424	0.515	0.001	0.001	−0.001
Gender × Sexual orientation	89.74	1	89.74	2.797	0.095	0.006	0.006	0.004
Gender × Relationship status	47.11	1	47.11	1.468	0.226	0.003	0.003	0.001
Sexual orientation × Relationship status	45.50	1	45.50	1.418	0.234	0.003	0.003	0.001
Gender × Sexual orientation × Relationship status	120.93	1	120.93	3.769	0.053	0.008	0.008	0.006
**Touch Avoidance—Strangers**								
Gender	2.0205	1	2.0205	0.24395	0.622	0.001	0.001	−0.002
Sexual orientation	8.2365	1	8.2365	0.99444	0.319	0.002	0.002	−0.000
Relationship status	0.5375	1	0.5375	0.06489	0.799	0.000	0.000	−0.002
Gender × Sexual orientation	0.0586	1	0.0586	0.00707	0.933	0.000	0.000	−0.002
Gender × Relationship status	10.1274	1	10.1274	1.22273	0.269	0.003	0.003	0.000
Sexual orientation × Relationship status	5.3802	1	5.3802	0.64958	0.421	0.001	0.001	−0.001
Gender × Sexual orientation × Relationship status	5.6249	1	5.6249	0.67912	0.410	0.002	0.002	−0.001

***Note***. Gender (male/female), sexual orientation (opposite-sex attraction/same-sex attraction), relationship status (single/in a relationship). * Significant at 0.05 level. ** Significant at 0.01 level. *** Significant at 0.001 level.

**Table 3:** Estimated marginal means (and standard errors) of the touch avoidance with same-sex friend score by gender, sexual orientation, and relationship status.

	Male		Female	
	Single	In a Relationship	Single	In a Relationship
	*EMM (SE)*	*EMM (SE)*	*EMM (SE)*	*EMM (SE)*
**Opposite-sex attraction**	14.80 (0.60)	14.82 (0.54)	10.25 (0.68)	10.56 (0.47)
**Same-sex attraction**	13.40 (0.94)	9.15 (1.14)	8.08 (1.09)	10.30 (0.92)

## Data Availability

The datasets used and/or analysed during the current study are available from the corresponding author on reasonable request.
